# Supplementary material for: Identification of putative reader proteins of 5-methylcytosine and its derivatives in Caenorhabditis elegans RNA
Source: Wellcome Open Res. 2022 Nov 17;7:282. [Version 1] doi: 10.12688/wellcomeopenres.17893.1 (PMC10354459; doi:10.12688/wellcomeopenres.17893.1)
Supplement: Peer review history

# Peer review: Reviewer response for version 1

**DOI:** 10.21956/wellcomeopenres.19826.r59586

**Published:** 2023-07-18

**Authors:** Anna Y Zinovyeva, Jeffrey Medley

**Affiliations:** 1 Kansas State University, Manhattan, Kansas, USA; 2 Division of Biology, Kansas State University, Manhattan, Kansas, USA

RNA modifications have gained much interest in the recent years as they become associated with important biological functions. 5-methylcytosine (m ^5^C) is an abundant and conserved post-transcriptional modification found on a variety of RNA molecules. Thus far, much effort has been placed on identifying m ^5^C modification sites and unraveling the biological significance of these modifications through “writer” loss-of-function experiments. More is currently known about the “writers” and “erasers” of the m ^5^C modifications than the “readers”, which could introduce specificity and unique biological effects through combinatorial modification plus target context.

 In “Identification of putative reader proteins of 5-methylcytosine and its derivatives in *Caenorhabditis elegans* RNA”, Navarro *et al.* takes advantage of *C. elegans*, with its excellent genetic tractability, to identify potential readers of m ^5^C modification and its derivatives. Identifying proteins that are responsible for reading specific RNA modifications is an important and valuable first step towards understanding the functional significance of distinct RNA modifications. This study aims to do this by identifying *C. elegans* proteins that preferentially interact with synthetic **RNA baits containing different chemical modifications. The mass spec identification of bait RNA-associated proteins is a great first-order approach and is thus well-conceived, with appropriate unmodified RNA oligonucleotides and beads-only controls. However, addressing two major areas would be needed to elevate this manuscript to its intended form as a community resource.

 1. The first key issue that needs to be addressed is demonstrating to the readers that the provided lists of putative modified oligo interactors are a solid starting point for follow up work. This can be achieved through addressing the following comments:

 Additional mass-spec data analysis should be presented to increase the scientific readers’ confidence in the identified lists of putative m ^5^C, etc. “readers”. Usage of the intensity-based absolute quantification (iBAQ) *median* across three (3) replicates could be wildly misleading if there is a high degree of variability among the replicates and if pairing of the data exists. Use of a median essentially reduces the analysis to a single replicate comparison between the experimental conditions. One way to bolster the confidence in the data would be to provide a principal component analysis (or analyses) of the proteomics data to demonstrate data consistency between control and m ^5^C (and derivatives) experimental conditions.   

 Figure 1 legend and manuscript text: Please define what you mean by “Proteins uniquely enriched in pulldowns”. Does that mean 1. proteins that were enriched 1.5 or 2-fold over control in each experiment, 2. proteins that were unique to modification type but not enriched over control, or 3. proteins that were 1.5 or 2-fold enriched over control and unique to m5C pulldown vs. other modifications? The reader assumption is that it’s likely the latter, however, Figure 1B highlights “uniquely enriched proteins” that appear to fail the 1.5 or 2-fold enrichment threshold. Please address this discrepancy. In addition, use of 1.5- or 2-fold lines on Figure 1B plots, as well as color-coding fold enrichment vs. uniqueness in the plots would further improve the clarity of the data analysis. Labeling highly changed/high confidence candidate readers on the plots (as well as ALY-1, ALY-2, etc.) would also provide context of these proteins’ enrichment for the reader. As shown, Figure 1B provides limited and unclear information.

 The expectation inherent to the described experimental approach is that the “reader” proteins are uniquely and specifically able to discriminate between m ^5^C RNA modification and its derivatives hm ^5^C, hm ^5^Cm, and f ^5^Cm. A discussion addressing the justifications for this expectation is important to set the stage for the reader to understand the appropriateness of the “uniquely binding” criteria of the co-precipitating proteins. A protein appears to be disqualified from being considered a candidate “reader” if it is not unique to any given modification derivative. Is there a precedent of this assumption and could this assumption perhaps erroneously eliminate “readers” capable of reading m5C and some of its derivatives? Further discussion on this is needed to ensure the manuscript audience can evaluate the validity of the approach used to qualify any given putative interactor as a potential “reader”. 

 In a similar vein of thought, the manuscript does not address the justifications for using the specific oligos and the three modification sites within each oligonucleotide. Thus, the manuscript would greatly benefit from a discussion of what previous data drove the design of the RNA oligonucleotides used in this study, as well as the justification for using the three (3) specific locations for m ^5^C within the oligos. Some discussion about how the baits were designed and potential limitations would be helpful to understand the significance of the findings. Given that many m ^5^C modification occurs on tRNAs, were the baits designed to mimic the tRNA structure (since the structural context is important)? Is there evidence that RNAs contain these specific modifications at multiple sites? If so, is it known whether modification “readers” can differentiate between molecules containing multiple modifications and single modifications? If single-modified baits were used, how might that impact the identified readers? It seems plausible that some “readers” might differentiate between single-stranded, double-stranded and loop modifications. An expanded discussion of some of these points would help clarify the significance and limitations of this study, necessary to identify potential avenues for further analysis.

 2. In Figure 3A, the residues required for RNA binding are highlighted, with the authors referring to Yang *et al.*, 2017 ^1^: “residues in ALYREF required for m ^5^C binding previously identified by Yang *et al.* are highlighted in red”. However, these residues do not appear to be defined in that study. According to Yang *et al*., 2017 ^1^, the same residues are described as being “the relatively conserved amino acids used for constructing mutants” (and have not each been validated as important for RNA binding as suggested in this study, with one potential exception of K171). The authors should also clarify the relevance of the Yang *et al*., 2019 ^2^ citation, as it appears to have been referenced in error.  

 In Figure 3A and 3D, numbering of amino acid positions would help orient manuscript readers to positions of functional domains and understand the take-home message. In Figure 3A, the highlighted residues appear to be different from the RNP1 and RNP2 motifs highlighted in Figure 3D. What is the basis for RNP1 and RNP2 being highlighted in Figure 3D as RNA binding domains? This should be clarified in both the manuscript text and figure legend. Further, these reviewers are not convinced that the provided alignment shows a “high degree of conservation”, as shown in Figure 3D. Usage of protein isoforms identical for the shown region can skew the Muscle analysis and more information is needed for why the authors deem the highlighted regions (RNP1 and 2) in Figure 3D as “important for nucleic acid binding”. Finally, the language should be softened to propose the highlighted “RNP” regions as potentially important, with qualifications that the highlighted regions have not been demonstrated to bind nucleic acids. Justifications for highlighting the proposed regions should still be provided.

 ALY-1/2 pulldowns/Western findings would be bolstered by providing all replicates in Supplemental data. In addition, language within the manuscript should be softened to avoid stating that pulldowns and Western unambiguously confirm ALY-1 and ALY-2 as m ^5^C readers.

**Additional comments:**

 These reviewers appreciate the list of putative interactive proteins provided as a resource in The Supplemental Table 1. However, the practical usability of that resource would be dramatically improved by including the *C. elegans* gene names. This would also allow the readers to independently assess the nature of the putative interacting proteins beyond the provided GO term analysis.

 Figure 3C: The legend should state that only the RRM domains of the proteins are shown. This will prevent manuscript readership from misinterpreting the shown predicted structures as structurally conserved across the entire protein length.

 Based on GO-term analysis, the authors claim that hm5C and f5C modifications show “exclusively germline related phenotypes”. However, one of the phenotypes for hm5C was sluggish, which seems unlikely to result from a germline defect.

 Please state in the Methods section how much protein was used per each pulldown experiment going into mass spectrometry. As written, it appears that only 2mg of protein has been used, an inadequate amount for most mass spectrometry experiments. Alternatively, please reference the literature that supports that 2mg of protein is sufficient for RP-LC mass spectrometry.

Is the work clearly and accurately presented and does it cite the current literature?

Partly

If applicable, is the statistical analysis and its interpretation appropriate?

Not applicable

Are all the source data underlying the results available to ensure full reproducibility?

Partly

Is the study design appropriate and is the work technically sound?

Partly

Are the conclusions drawn adequately supported by the results?

Partly

Are sufficient details of methods and analysis provided to allow replication by others?

Partly

Reviewer Expertise:

RNA biology, C. elegans, RNA pulldown techniques

We confirm that we have read this submission and believe that we have an appropriate level of expertise to confirm that it is of an acceptable scientific standard, however we have significant reservations, as outlined above.

## References

[1] : 5-methylcytosine promotes mRNA export - NSUN2 as the methyltransferase and ALYREF as an m5C reader. *Cell Res* .2017;27(5) : 10.1038/cr.2017.55 606-625 10.1038/cr.2017.55 28418038PMC5594206

[2] : RNA 5-Methylcytosine Facilitates the Maternal-to-Zygotic Transition by Preventing Maternal mRNA Decay. *Mol Cell* .2019;75(6) : 10.1016/j.molcel.2019.06.033 1188-1202.e11 10.1016/j.molcel.2019.06.033 31399345

# Peer review: Reviewer response for version 1

**DOI:** 10.21956/wellcomeopenres.19826.r61033

**Published:** 2023-07-11

**Authors:** Carolyn Phillips

**Affiliations:** 1 University of Southern California, Los Angeles, California, USA

m ^5^C is a conserved RNA modification that has been detected in tRNAs, rRNAs, and mRNAs from other systems that has roles such as promoting tRNA stability and translational fidelity. In *C. elegans*, m ^5^C is non-essential but nonetheless prevalent in rRNAs and tRNAs, as shown by the same research group in previous work (Navarro et al EMBO 2020). mRNA modification appears to be less common. Loss of m ^5^C from *C. elegans* leads to developmental and fertility defects as well as a decrease in translational efficiency. Here, Navarro et al seek to identify readers of the m ^5^C modification in *C. elegans*. Only two m ^5^C readers have been identified to date in any organism, so identification of more candidate proteins is an important question. To this end, Navarro *et al*, synthesize RNA baits modified with m ^5^C and derivatives, incubate the baits with RNA lysate, and following pulldown, perform mass spec for interacting proteins. A significant number of candidate readers were identified, including orthologs of ALYREF, a previously known m ^5^C reader, which were then further validated by m ^5^C RNA pulldown and western blot. Further analysis will be needed to parse the false positives from the true m ^5^C readers and to determine the biological function of each protein.

 Comments –

- RNA baits were designed in three structural contexts, single-strand, double-strand and loop – but it is not discussed in the results whether the identified m ^5^C candidate readers were found using one or all three of these structures. And were unmodified baits also used in all three structural contexts?
- The text indicates that candidates were limited to those that bound only one of the modified RNA baits, but it is not discussed whether the known readers are unable to bind the m ^5^C variants. Is it possible that interesting candidates would have been eliminated at this step? At a minimum, the full list of mass spec interactors, including those found binding multiple m ^5^C variants should be provided.
- Figure 3A and text “Protein alignments show that some of these residues are conserved in ALY-1 and ALY-2” – Of the residues highlighted in the red box, several are weakly conserved or not conserved at all. It is not discussed whether the loss of these critical residues will likely affect the ability of these proteins to bind the m ^5^C modification.
- In the last sentence of the results, the word “bona fide” should be removed since these interactions have not yet been validated.

Is the work clearly and accurately presented and does it cite the current literature?

Yes

If applicable, is the statistical analysis and its interpretation appropriate?

Partly

Are all the source data underlying the results available to ensure full reproducibility?

Partly

Is the study design appropriate and is the work technically sound?

Yes

Are the conclusions drawn adequately supported by the results?

Yes

Are sufficient details of methods and analysis provided to allow replication by others?

Partly

Reviewer Expertise:

C. elegans RNA silencing

I confirm that I have read this submission and believe that I have an appropriate level of expertise to confirm that it is of an acceptable scientific standard.

# Peer review: Reviewer response for version 1

**DOI:** 10.21956/wellcomeopenres.19826.r53385

**Published:** 2022-12-09

**Authors:** Matthias Ronald Schaefer

**Affiliations:** 1 Centre for Anatomy and Cell Biology, Division of Cell-and Developmental Biology, Medical University of Vienna, Vienna, Austria

Besides mapping chemical modifications in RNA, the vast majority of epitranscriptomics studies is currently reporting on associations between the function of a gene (writer, reader, eraser) and a multitude of observable phenotypes. These approaches are usually very simplistic and classically involve gene knockdowns or even knockouts, followed by the description of phenotypic changes, many of which seem to be rather pleiotropic resulting in little insight which of the RNA substrates of a particular writer, reader or eraser protein has contributed to the observed changes. Interestingly, even though mapping studies have provided positional clues about the occurrence of particular modifications, there seems to be little incentive in following up on assigning functional impact of specifically modified nucleotides in specific RNAs on a particular trait and/or phenotype.

 It is accepted that RNA modifications are “written” onto RNA sequences by a staggering number of dedicated enzymes. While non-coding RNAs are often stoichiometrically modified with a great number of different RNA modifications (i.e., tRNAs) , mRNAs, while being modified with a few different RNA modifications, carry only sub-stoichiometric levels of a particular modification at specific nucleotides. It follows that modified nucleotides in mRNA are not only rare within a transcript (1-3 modified nucleotides per 1000 nucleotides when averaged over all mRNAs for the most abundant mRNA modification, m6A) but also stoichiometrically low (e.g., about 20% m5C can be detected at a given position based on RNA bisulfite sequencing). This is an important and seemingly overlooked conundrum in the field, since it begs the question how a few nucleotides within thousands of nucleotides, and those at every 5 ^th^ to 10 ^th^ mRNA molecule should account for physiologically meaningful impact on the biology of a cell.

 A solution for this presents the concept of reader proteins. This concept has been put forward by epitranscriptomic influencers borrowing heavily from the notion of epigenetic readers. If there are only a few RNA modifications within a sea of transcripts, then proteins binding preferentially to this modified nucleotides (readers) might make the difference. Is this concept correct? Do experimentally validated data support the assumption that a few RNA modifications will make a difference through interpretation of particular proteins?

 In the last years, especially the m6A-focused field has reported on the identification of various m6A readers. A few attempts also exist in the public record that identified readers of other RNA modifications, including m5C. Notably, much of the current identification and characterization of these RNA modification readers relied on in vitro pull-down experiments using either defined (synthetic) RNA baits (rather than those identified by epitranscriptomic mapping strategies) combined with mass spectrometry or RNA-IPs (and derivatives of the method) to identify RNA substrates of specific reader proteins. From these reports one can deduce that all readers of specific RNA modifications also bind to unmodified RNA sequences. This begs the question if readers bind with lower Kd to modified RNAs than to unmodified RNAs? Curiously, very little has been published reporting on actual interaction parameters for specific readers and their substrates, which is common when studying enzymes or protein-substrate interactions. Instead, fixed end-point assays with readouts based on RNA sequencing or western blotting are currently substituting for deriving quantitative and kinetic parameters from the identified interactions, which would be needed to better understand the biology of putative readers of RNA modifications.

 This manuscript by Navarro et al., 2022 is therefore welcome for attempting to identify reader proteins for m5C as potential mediators of RNAs modified with this chemical modification.

 Assuming that C. elegans contains readers for the m5C modification and its oxidized derivatives, the authors use an in vitro RNA pull-down approach with differently modified RNA baits to identify proteins differentially binding to m5C, hm5C, hm5m and f5C. Setting rather tight cut-offs for enrichment of controls, they report on numbers and GO annotations of the identified proteins. Since they found the orthologues of a previously identified human m5C reader protein (ALYREF) in their data, they repeated the pull-down experiment with m5C-modified oligos and identified both worm orthologues of ALYREF to bind preferentially to m5C-modified oligos.

 This reviewer accepts that Wellcome Open Research publishes articles reporting any original research that has been funded (or co-funded) by Wellcome, without editorial bias including papers reporting single findings, replications studies, and null results or negative findings. This reviewer will therefore only assess the validity of the article content, rather than the novelty or interest levels.

Comments:

1. This manuscript cannot be disconnected from Navarro et al., 2020 ^1^, which reported on only 188 positions in the worm transcriptome as putative m ^5^C sites. Their final conclusion about m ^5^C in C. elegans: “In summary, we found no evidence of a widespread distribution of m ^5^C in coding transcripts. To investigate whether the presence of common characteristics could support a subset of the aforementioned 188 positions as bona fide methylated sites, we performed gene ontology, motif search, genomic localisation and secondary structure analyses on these transcripts and sites; however, no significant shared features were found. While our data do not completely rule out the existence of m5C methylation in mRNAs, it demonstrates that this mark cannot be detected in high stoichiometry in C. elegans, as observed in tRNAs and rRNAs.” These published results hints towards the fact, that putative readers of m5C in worms will read this modification likely in tRNA and rRNA but not in mRNA. This reviewer suggests to place a reminder of their findings into the introduction of the manuscript, since the current version will be read by many scientists, eager to put meaning into exceptions, as proof that C. elegans contains m5C in mRNAs which affect its adaptability to high temperatures.
2. The RNA bait is depicted as a secondary structure in Figure 1a. Please specify in the M&M, how you achieved formation of such structures before performing pull-down experiments with protein extracts.
3. PAGE 4: “To increase the chances to identify bona-fide modification readers…” Since synthetic and not naturally occurring modified cytosine-containing RNA oligos were used, this reviewer would not call the “to-be-identified readers” bona fide (meaning genuine, true).
4. Figure 1b: To this reviewer, mustard is not the color of choice to highlight enriched proteins. Please make this readable. Please, indicate what the vertical and horizontal lines of proteins should signify. Based on the Figure legend, this reviewer would expect them to be all in mustard since they are clearly enriched of unmodified oligo. However, these identities are marked by all colors.
5. PAGE 6: “Yang *et al.* identified amino acid residues on ALYREF required for binding to m ^5^C (Yang *et al.*, 2019 ^2^). Protein alignments show that some of these residues are conserved in ALY-1 and ALY-2 (Figure 3A).” Yang et al., reported on exactly one (1) amino acid (K171) that seemed to be required for binding of ALYREF to m5C-modified RNA oligos. Please, correct this sentence. Figure 3a shows that exactly this lysine (human K171) is a glutamine (Q) in both worm aly-1 and -2. Even though a few other residues in the shown sequence are conserved, none really matters since lysine within the sequence of AMKQ in human ALYREF is the only residue to be important as published by Yang et al., 2019. Spoiler alert for readers of this review: in S4G of Yang et al., 2019, exactly at the level of ALYREF (with the K171A mutation), the binding to the 5mC oligo is obscured by a blind spot (air bubble?), which makes it questionable if that the signal is really reduced.
6. Figure 3a: “*” represents identical amino acids while “:” and “.” represent amino acids with strongly and weakly similar properties, respectively.” Please indicate, which protein substitution/similarity index was used to support this statement.
7. Figure 3b: “Anti-FLAG western blotting (left) showing enrichment of ALY-1 and ALY-2 following pulldown using m ^5^C-modified RNA oligos and relative quantification (right). Mean ± SD, quantification performed using ImageJ, n = 2 independent biological replicates.“ Provided raw blots at https://data.mendeley.com/datasets/k6z3c5xftr/3 do not show replicates of pull-down followed by aly-2-FLAG identification. Please provide, or state that the data come from a single experiment. A comment for interested readers: quantification of overexposed inputs is notoriously difficult and likely not possible. Hence, either a titration curve on input protein or lower exposure would convince this reviewer of the stated enrichment factors using density measurements.
8. “Amino acid residues shown to be important for m5C binding in ALYREF also reside in the RNA recognition motif (RMM) domain, which is a known RNA-binding domain (Yang et al., 2017 ^3^).” same overstatement as before. Yang et al., 2017 only reported on K171, which lies at the border of the RRM and is not conserved in any of the proteins shown in Figure 3. Please, rewrite this statement to avoid that AI-based manuscript writing picks up on it and propagates this inconsistency.
9. Given that aly orthologues of ALYREF were “only” 1.9x enriched to bind to m5C over C-oligo, what about the other binders listed in Supp Table 1? Provided that m5C is likely present in tRNA and rRNAs in C. elegans, how many of the identified proteins in the experiments bind to tRNAs or rRNA?

Is the work clearly and accurately presented and does it cite the current literature?

Partly

If applicable, is the statistical analysis and its interpretation appropriate?

Not applicable

Are all the source data underlying the results available to ensure full reproducibility?

No

Is the study design appropriate and is the work technically sound?

Partly

Are the conclusions drawn adequately supported by the results?

Partly

Are sufficient details of methods and analysis provided to allow replication by others?

Partly

Reviewer Expertise:

RNA biology

I confirm that I have read this submission and believe that I have an appropriate level of expertise to confirm that it is of an acceptable scientific standard, however I have significant reservations, as outlined above.

## References

[1] : Translational adaptation to heat stress is mediated by RNA 5-methylcytosine in Caenorhabditis elegans. *EMBO J* .2021;40(6) : 10.15252/embj.2020105496 e105496 10.15252/embj.2020105496 33283887PMC7957426

[2] : RNA 5-Methylcytosine Facilitates the Maternal-to-Zygotic Transition by Preventing Maternal mRNA Decay. *Mol Cell* .2019;75(6) : 10.1016/j.molcel.2019.06.033 1188-1202.e11 10.1016/j.molcel.2019.06.033 31399345

[3] : 5-methylcytosine promotes mRNA export - NSUN2 as the methyltransferase and ALYREF as an m5C reader. *Cell Res* .2017;27(5) : 10.1038/cr.2017.55 606-625 10.1038/cr.2017.55 28418038PMC5594206
